# Dynamic nuclear polarization at 40 kHz magic angle spinning†
†Electronic supplementary information (ESI) available: Experimental details, with supplementary tables and figures. See DOI: 10.1039/c6cp00839a



**DOI:** 10.1039/c6cp00839a

**Published:** 2016-03-14

**Authors:** Sachin R. Chaudhari, Pierrick Berruyer, David Gajan, Christian Reiter, Frank Engelke, Daniel L. Silverio, Christophe Copéret, Moreno Lelli, Anne Lesage, Lyndon Emsley

**Affiliations:** a Institut de Sciences Analytiques , Centre de RMN à Très Hauts Champs , Université de Lyon (CNRS/ENS Lyon/UCB Lyon 1) , France . Email: anne.lesage@ens-lyon.fr; b Bruker Biospin , 76287 Rheinstetten , Germany; c ETH Zürich , Department of Chemistry , Vladimir Prelog Weg 1-5 , CH-8093 Zürich , Switzerland; d University of Florence , Chemistry Department , Magnetic Resonance Center , 50019 Sesto Fiorentino (FI) , Italy; e Institut des Sciences et Ingénierie Chimiques , Ecole Polytechnique Fédérale de Lausanne (EPFL) , CH-1015 Lausanne , Switzerland . Email: lyndon.emsley@epfl.ch

## Abstract

DNP-enhanced solid-state NMR spectroscopy under magic angle spinning (MAS) is rapidly developing into a powerful analytical tool to investigate the structure of a wide range of solid materials, because it provides unsurpassed sensitivity gains. Most developments and applications of DNP MAS NMR were so far reported at moderate spinning frequencies (up to 14 kHz using 3.2 mm rotors). Here, using a 1.3 mm MAS DNP probe operating at 18.8 T and ∼100 K, we show that signal amplification factors can be increased by up to a factor two when using smaller volume rotors as compared to 3.2 mm rotors, and report enhancements of around 60 over a range of sample spinning rates from 10 to 40 kHz. Spinning at 40 kHz is also shown to increase ^29^Si coherence lifetimes by a factor three as compared to 10 kHz, substantially increasing sensitivity in CPMG type experiments. The contribution of quenching effects to the overall sensitivity gain at very fast MAS is evaluated, and applications are reported on a functionalised mesostructured organic–inorganic material.

## Abstract 2

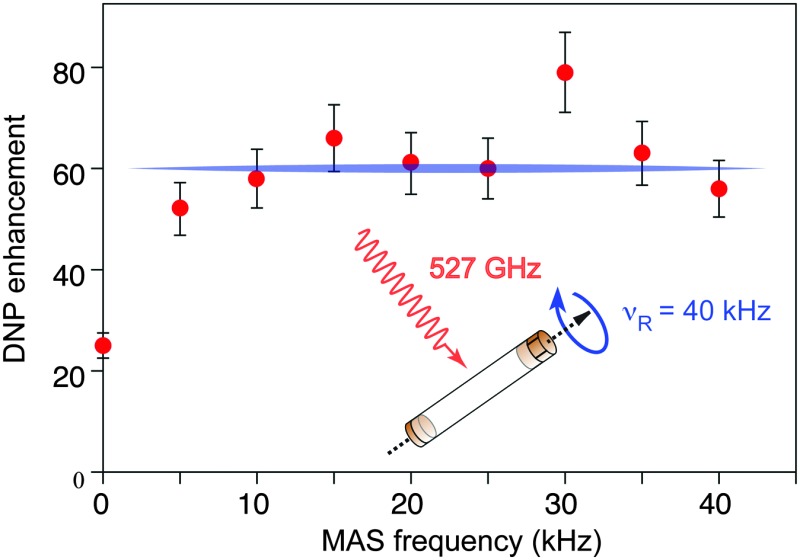
DNP-enhanced solid-state NMR spectroscopy is shown to maintain performance over a wide range of sample spinning rates up to 40 kHz.

## Introduction

Over the last few decades, magic-angle spinning (MAS) solid-state NMR has developed into an essential analytical tool to investigate the structure and dynamics of chemical and biological systems.^1^ While it can provide in many cases unprecedented insights into atomic-scale structures, solid-state NMR suffers from low sensitivity, which strongly limits its application fields. One intriguing possibility to increase the sensitivity of solid-state MAS NMR experiments is dynamic nuclear polarization (DNP).^2^ In a DNP experiment, the large polarization of unpaired electrons is transferred upon microwave (μw) irradiation to surrounding nuclei providing a maximum theoretical signal enhancement (*ε*_max_) of |*γ*_e_/*γ*_n_,| where *γ*_e_ and *γ*_n_ are the gyromagnetic ratios of the electron and of the polarized nucleus, respectively (*ε*_max_ ∼ 658 for ^1^H). Although the principles of this effect have been known for many decades,^2a,3^ its transposition to modern high-resolution NMR of solids at magnetic fields of 5 T or higher has become possible only relatively recently, with the introduction of gyrotron sources capable of delivering high-power high-frequency microwaves,^4^ and of cryogenic MAS probes for experiments at 100 K or lower temperatures. In this respect, in the past few years, high-field MAS DNP-enhanced solid-state NMR spectroscopy has made great progress^5^ and has been implemented successfully on a wide variety of systems, ranging from porous and non-porous materials,^6^ colloids,^7^ molecular organic solids including pharmaceuticals,^8^ polymers,^9^ membrane proteins, fibrillar aggregates, biomaterials or cells,^10^ thus gaining more and more importance.

Continuous wave MAS DNP can proceed *via* several mechanisms including the solid-effect (SE), the cross-effect (CE), and the Overhauser effect (OE).^4b,11^ Many aspects of these mechanisms have recently received attention in order to optimize DNP performance. Using for example the binitroxides in the AMUpol^12^ and TEKpol^13^ families, whose structure has been specifically designed to optimize the CE DNP process, signal enhancement factors of over two orders of magnitude (up to 250 in bulk solutions, and up to 500 in optimized samples^13*b*^) are now routinely obtained at magnetic fields of 5–9.4 T and sample temperatures of *ca.* 80–105 K. Enhancements of between 20 and 75 have been recently achieved in frozen bulk solutions at 18.8 T (800 MHz) using BDPA^11f,14^ or mixed biradicals, in which a nitroxide and a narrow-line radical are chemically tethered.^15^ With specially optimised solvent/radical formulations significant enhancements have been achieved with CE and OE DNP near room temperature.^14^

Despite this spectacular progress, and although sample spinning is an intrinsic part of these experiments, the sample spinning rate itself has so far received little attention. Indeed, with the notable exception of experiments reported recently at spinning speeds up to 25 kHz using He gas,^16^ high-field DNP NMR spectroscopy is currently carried out almost exclusively using 3.2 mm rotors and with maximum MAS frequencies of ∼15 kHz. However, the advent of MAS probes of increasingly smaller diameter and capable of spinning samples ever faster (up to >100 kHz today^17^) has been one of the key driving forces for the spectacular progress that has been made in the last decade or so. These developments were triggered by the improvement in spectral resolution and coherence lifetimes engendered by faster sample spinning, which averages out orientation-dependent interactions in an increasingly efficient way. DNP-enhanced solid-state NMR experiments are expected to benefit along similar lines through the introduction of cryogenic MAS probes with smaller rotor diameters.

However, several questions regarding the relevance of developing DNP solid-state NMR under faster magic angle spinning remain open. They concern for example the impact of very fast MAS on the DNP mechanisms themselves, on the spin diffusion process that relays the enhanced polarization through the sample, on the saturation efficiency of the EPR line, on the sample temperature factor, or on the so-called quenching effects^18^ that are a major source of signal loss in DNP experiments. All these elements will influence the effective sensitivity enhancement factor at fast MAS, and the nature of the spinning frequency dependence of the DNP enhancement is still the subject of speculations.^11c–e,g,h^

Based on the original descriptions of the cross-effect,^19^ the most detailed and recent predictions for MAS CE DNP have been provided by theoretical and computational studies from Thurber and Tycko^11*c*^ and from Mentink-Vigier *et al.* ^11d,h^ They showed that, as the Hamiltonian of the spin system becomes periodically time dependent, several types of energy level crossing occur when the Larmor frequencies of the two electrons match the CE condition, the μw irradiation frequency, the SE condition, or the Larmor frequency of the second electron. Depending on many parameters, including among the others the spinning frequency, the electron–electron dipolar coupling, the magnetic field and the electron relaxation times, the rate of passage through the energy level crossing changes. This leads to polarization transfer with varying degrees of adiabaticity and to subtle changes in electron and nuclear populations. After a rapid increase at MAS frequencies below 2 kHz, numerical simulations predict a progressive decrease of the nuclear polarization with increasing spinning frequency.^11c,d,g^ This is in agreement with experimental observations that reported a decrease of the enhancement factor with increasing MAS rates.^4c,11d,g,h^ However, note that this decrease in *ε* was partly explained by the increase in sample temperature upon faster spinning^4*c*^ and was not observed in temperature-controlled experiments on bulk solutions,^13*a*^ where roughly constant DNP enhancement factors were observed over a 3 to 15 kHz MAS range. Note also that the dependence of *ε* on the spinning frequency is predicted to depend on the electron relaxation times. In particular long relaxation times are expected to lead to little or no decrease in *ε* with increasing spinning rate, which was likely the case in the study of Zagdoun *et al.* ^13*a*^

Beyond the enhancement factor *ε*, a range of effects leads to changes in the overall sensitivity of the NMR experiment. They include paramagnetic “bleaching” of the NMR signals due to the proximities of some of the nuclei to paramagnetic centers, increased linewidths for some samples at low temperature, increased thermal polarization at low temperature, shorter nuclear *T*_1_ values in the presence of radicals, as well as other factors that have been discussed and quantified in several papers.^18^ Most of these factors will also depend on the spinning frequency.

In addition to these elements, Thurber and Tycko have recently discovered the presence of a depolarization mechanism for the nuclear states.^11*e*^ This effect occurs through CE DNP induced by MAS alone in frozen solutions that are paramagnetically doped with nitroxide-based radicals, in the absence of microwave irradiation. It adds to paramagnetic bleaching, and can lead to a potentially large reduction of signal intensity at low temperatures (up to a factor 6 reduction under MAS at 24 K with respect to undoped solutions).^11*e*^ CE nuclear depolarization by a factor estimated to be 20% and 60% has been observed for, respectively, TOTAPOL and AMUpol at 110 K and 400 MHz.^11*g*^ Numerical simulations predict that the depolarization process will increase with the spinning frequency. The current models however are dependent on many parameters including the *g*-tensor, the electron relaxation times, the electron–electron dipolar coupling, the electron–nuclear hyperfine coupling or the effective microwave power. These parameters are not all accurately known under the experimental conditions routinely used in MAS DNP and it is thus hard to safely predict what would be the effective overall DNP enhancement for MAS frequencies >15 kHz. In summary, it is thus currently not clear what to expect at higher spinning rates.

In the present work, we contribute to this debate by presenting an experimental investigation of DNP enhancements at up to 40 kHz MAS. Using a prototype 1.3 mm DNP probe operating at 18.8 T and 100 K, we determine the effect of fast MAS (up to 40 kHz) on CE DNP enhancements. We first report that the enhancement remains roughly constant in a spinning frequency range of 10 to 40 kHz, with *ε* values reaching a plateau of around 60. We then show that, as predicted and previously observed for lower spinning regimes, quenching effects induce an ever-larger signal loss with increasing MAS. However at 40 kHz spinning rates, a significant fraction of the sample, roughly 45%, still contributes to the NMR signal in ^13^C CPMAS experiments. Spinning at 40 kHz is also shown to increase ^29^Si coherence lifetimes in hybrid materials a factor between 6 and 10 as compared to 10 kHz, substantially increasing sensitivity in CPMG type experiments. We show that this allows for the measurement of multi-dimensional spectra on surface species under fast magic angle spinning using the DNP SENS approach.

## Results and discussion

**Fig. 1:**
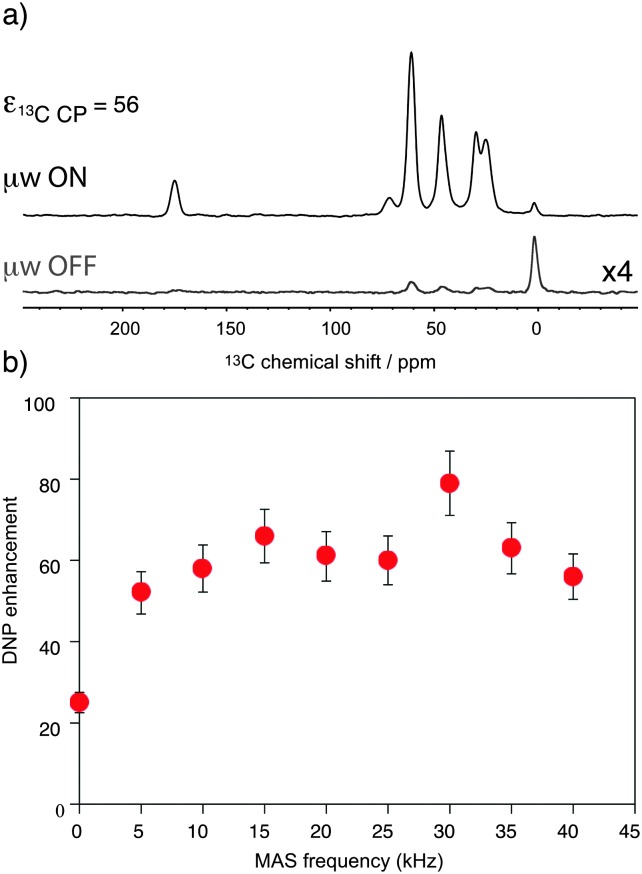
(a) ^13^C CPMAS NMR spectra of uniformly ^13^C-labeled proline (0.25 M proline in glycerol-d_8_/D_2_O/H_2_O, 60 : 30 : 10 volume ratio containing 10 mM AMUpol) at 18.8 T and 40 kHz magic angle spinning. The spectra were recorded with (upper spectrum) or without (lower spectrum) μw irradiation at 527 GHz to induce DNP. The resonance at 0 ppm corresponds to the silicon plug insert present at the top of the rotor. For all experiments, the main magnetic field was swept so that the fixed frequency of the gyrotron yielded the maximum enhancement for AMUpol. (b) MAS frequency dependence of the DNP enhancements measured from ^13^C CPMAS experiments measured on the proline resonances. A small amount of solid KBr was added (see the details in the ESI†) to the rotor to monitor sample temperature by measuring the ^79^Br *T*_1_ relaxation time.^20^ A sample temperature of 115 K ± 3 K was maintained over the whole spinning range for both microwave (μw) on and off experiments. The reported enhancements correspond to the mean value of the enhancement factors measured on the 5 carbon-13 resonances of proline. All measurements were performed with a microwave power of 16 W at the probe.

**Fig. 2:**
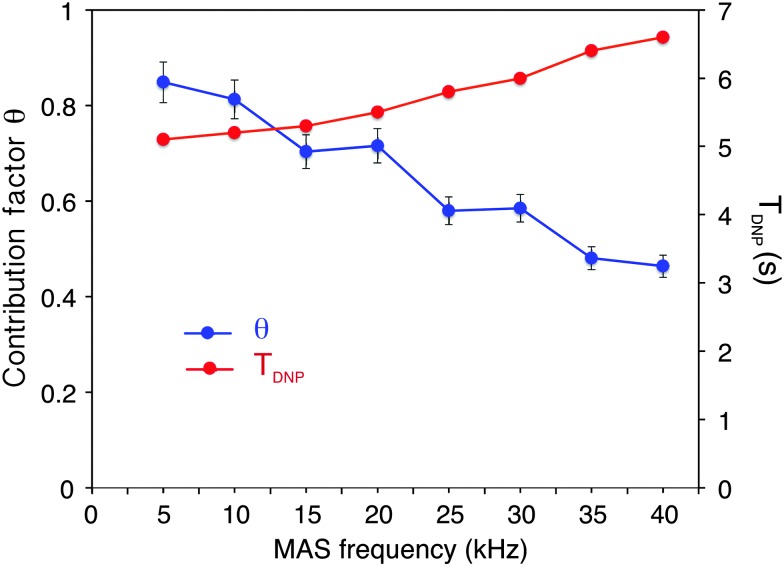
Blue circles: measured contribution factor *θ* to ^13^C CPMAS signal intensity as a function of the spinning frequency. Red squares: ^1^H *T*_DNP_ measured with a standard saturation recovery sequence followed by echo detection under microwave irradiation. The measurements were done at 18.8 T with solutions of 2-^13^C-labeled glycine (0.5 M glycine in glycerol-d_8_/D_2_O/H_2_O, 60 : 30 : 10 volume ratio containing or not 10 mM of AMUpol) as described in the main text. A constant sample temperature of 115 K ± 3 K was maintained over the whole spinning range. CP conditions were optimized for each spinning frequency and were kept the same for the two sets of experiments recorded on frozen solutions with and without the polarizing agent.

Fig. 1 shows the observed ^13^C DNP enhancement of uniformly ^13^C-labelled proline in a bulk water/glycerol (D_8_-glycerol/D_2_O/H_2_O; 60 : 30 : 10) solution containing 10 mM AMUpol as a function of the sample spinning frequency. These enhancements were obtained from temperature-controlled ^13^C cross-polarization (CP) experiments (Fig. 1a) using 1.3 mm zirconia rotors. Sample temperatures were adjusted to the same value at each spinning speed using the ^79^Br *T*_1_ relaxation time of KBr^20^ from one sample spinning speed to another, as well as between the μw on and off experiments. Fig. 1b shows that the enhancement factor first increases from nearly static (400 Hz) (*ε*_13C CP_ = 25) to 10 kHz MAS (*ε*_13C CP_ = 58), as expected for nitroxide-based radicals and previously reported a number of times.^4c,11d,g,13a^ We then observe that the enhancement does not vary too much over the spinning frequency range of 10 to 40 kHz. With an unexplained exception for the point at 30 kHz spinning frequency, enhancement factors of between 56 and 66 were observed over the range. These values are significantly higher (by about a factor 2) than enhancement factors obtained in a 3.2 mm sapphire rotor at 10 kHz MAS and 18.8 T under otherwise identical experimental conditions (values typically around 30, see Fig. S7, ESI†). In particular, high enhancement is preserved at 40 kHz MAS. This result suggests that smaller volume rotors and the smaller overall dimensions of the 1.3 mm MAS turbine and stator system relative to the microwave beam diameter as compared to larger diameter MAS systems may lead to an improved average microwave distribution in the sample and thus to higher signal amplification factors. We also recorded the *T*_DNP_ build up time as a function of MAS rate (Fig. 2, red curve) and we observed, as expected, that it linearly increases with the spinning frequency.

Note that using a 1.3 mm sapphire rotor, an enhancement factor *ε*_13C CP_ of 80 (see Fig. S8, ESI†) was obtained at a spinning frequency of 20 kHz (which was the maximum MAS rate that could be achieved using this rotor). This corresponds to an increase of 34% with respect to a zirconia rotor, in line with the fact that sapphire favors a better μw penetration.^13*b*^ To the best of our knowledge, this is the highest MAS DNP enhancement so far reported at 18.8 T. As discussed above, while some groups previously observed a drop in the CE enhancement factors with MAS frequencies up to 12 kHz at 9.4 T and 100 K, and while recent simulations and theoretical calculations allow for either a drop or a plateau,^11c,d^ the data here showing a plateau are in line with previous observations in temperature-regulated experiments done on bulk solutions containing bTbK derivatives.^13*a*^

As mentioned above and discussed by several groups,^18b–f^ *ε*_13C CP_ does not represent the overall sensitivity gain provided by a DNP experiment, and many factors have to be taken into account to evaluate the overall enhancement as compared to a “dry powder at room temperature,” one of which is the dependence of signal quenching on the spinning frequency. Quenching encompasses effects that include both signal losses due to direct paramagnetic bleaching and to depolarization effects. As discussed previously, both effects are expected to depend on MAS. Interestingly, paramagnetic bleaching should decrease with increasing spinning rate, whereas CE nuclear depolarisation is predicted (and has been observed) to increase with increasing spinning rates.^11e,g^ Fig. 2 shows the fraction *θ* of nuclei contributing to the NMR signal as a function of the MAS frequency from 5 to 40 kHz MAS.

This contribution factor was measured as described previously in ref. 18*b*, using samples of 2-^13^C-labelled glycine in bulk water/glycerol solutions containing, or not, AMUpol (at a concentration of 10 mM) and calculated as the ratio of the integrated intensities (II) of the CH_2_ resonance in ^13^C CPMAS spectra recorded in the absence of μw irradiation for solutions with and without AMUpol:
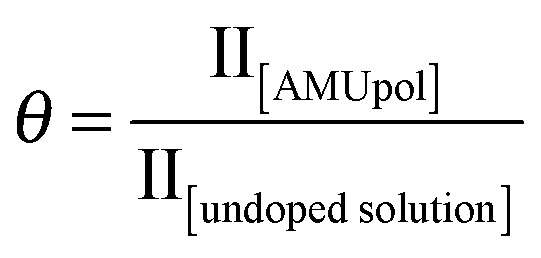
We observed that the contribution factor progressively decreases with increasing spinning frequencies, from 85% at 5 kHz to 46% at 40 kHz. Note that in addition to the paramagnetic bleaching and the depolarization effects mentioned above, *θ* also encompasses potential differences in CP efficiency between doped and undoped samples (for example due to differences *T*_1ρ_). Taking these values of *Θ* into account the enhancement *ε*_13C CP_ of 56 observed at 40 kHz MAS translates into a similar overall sensitivity enhancement factor of *Σ*_C CP_ = 55 when taken together with the faster relaxation due to the presence of the radical (here *Σ*_C CP_ was calculated as reported in ref. 18*b* using *T*_DNP_ and *T*_1_ values of, respectively, 6.5 s and 31 s for the doped and undoped solutions). This corresponds to an overall enhancement factor of *Σ*†C CP = 168 if we also consider the Boltzmann factor between a 100 K DNP experiment and an ordinary room temperature NMR experiment. This is a sizeable overall enhancement factor, which will allow the implementation with high sensitivity of NMR experiments that benefit from fast MAS.

**Fig. 3:**
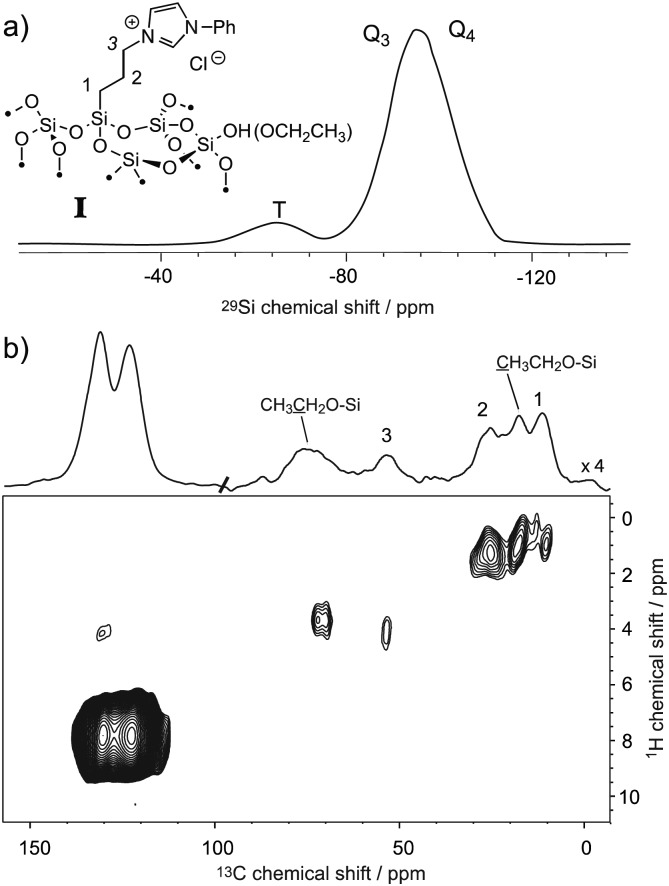
(a) One-dimensional natural abundance ^29^Si DNP-SENS CP-CPMG spectrum of **I**, recorded at 18.8 T (800 MHz) and 40 kHz MAS at a sample temperature of ∼115 K. The sample was impregnated with a solution of 10 mM AMUpol in 90 : 10 D_2_O/H_2_O, and transferred into a 1.3 mm rotor. During acquisition, SPINAL-64 decoupling^22^ was applied with a rf amplitude of 130 kHz. The cross-polarization (CP) contact time was 3 ms and the number of scans was 256 (total acquisition time 17 minutes). The CP-CPMG spectrum is shown in its echo reconstructed form and was obtained by adding up the whole echoes of the FIDs in the time domain, followed by Fourier transform and application of a first-order phase correction. A total of 60 echoes were acquired. Up to 120 echoes could be acquired (Fig. S13, ESI†). (b) Contour plot of a two-dimensional ^1^H–^13^C spectrum of **I** recorded with DNP at 18.8 T (800 MHz) and 40 kHz MAS. A total of 38 *t*_1_ increments of 40 μs with 256 scans each were recorded. The CP contact time was 700 μs, and the polarization-buildup interval was 4 s. The total experimental time was 10.8 hours. SPINAL-64 heteronuclear decoupling was applied during *t*_2_ with an rf amplitude of 100 kHz. During *t*_1_, eDUMBO-1_22_^23^ homonuclear decoupling was applied with an rf amplitude of 150 kHz. A scaling factor of 0.56 was applied to correct the ^1^H chemical shift scale.

**Fig. 4:**
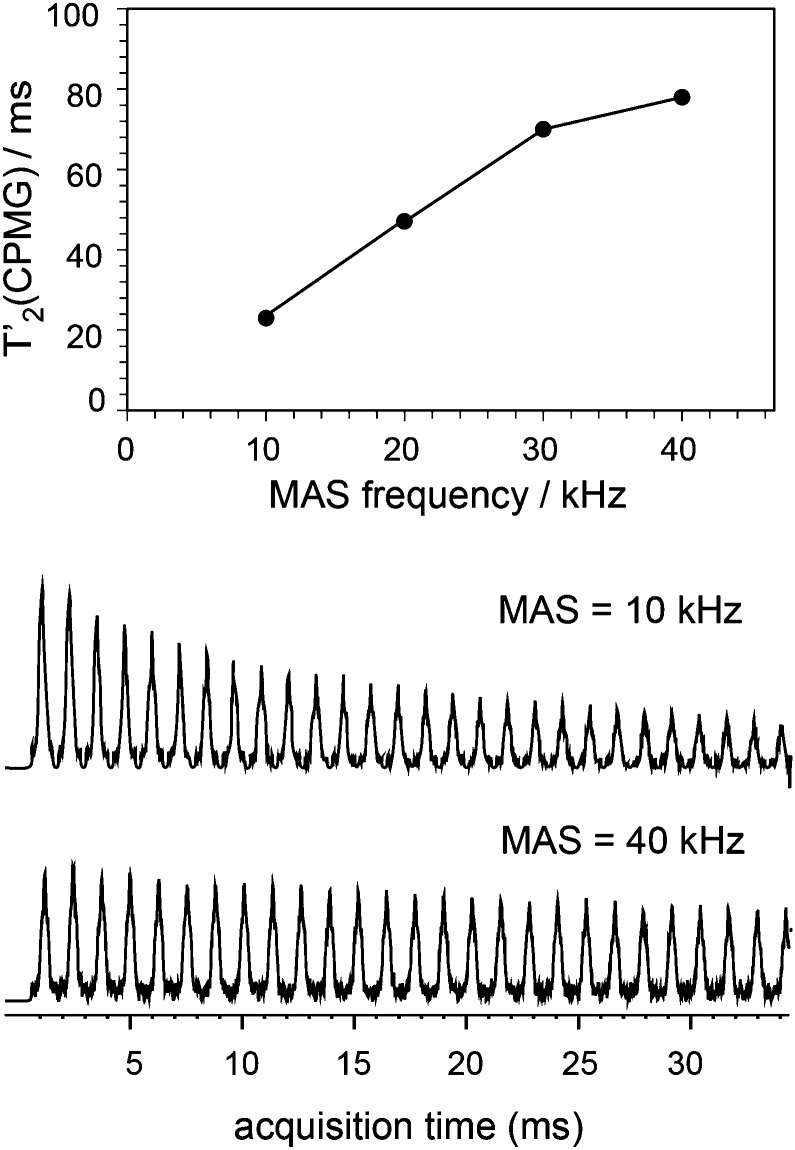
Plot of *T*_2_′_CPMG_ of Q_3/4_(^29^Si) sites as a function of the spinning frequency together with the free induction decays (FIDs) of ^1^H–^29^Si CP/CPMG experiments recorded at 10 and 40 kHz MAS frequency. SPNAL-64 decoupling at the 130 kHz RF field was applied during acquisition. The other experimental details were the same as those given in the caption of Fig. 3a.

The ability to enhance surface NMR signals in materials science is one of the most promising applications of DNP. This approach known as DNP surface enhanced NMR spectroscopy (DNP SENS) usually uses incipient wetness to impregnate porous or particulate materials with a radical containing solution.^5*a*^ As an example of the applicability of fast MAS DNP to actual substrates and not just bulk frozen solutions, Fig. 3 shows fast spinning DNP-SENS spectra of an organic–inorganic functionalized mesostructured material (**I**) at natural isotopic abundance (see the ESI† for details of the synthesis of the material). The material was impregnated with a D_2_O/H_2_O solution containing 10 mM AMUpol. A proton enhancement, measured on the solvent peak, of 16 was obtained over the whole spinning frequency range (from 5 to 40 kHz MAS, see Fig. S9, ESI†). Fig. 3a shows the ^29^Si DNP-SENS spectrum of **I** at 40 kHz MAS. In particular the combination of fast MAS with high-power heteronuclear decoupling leads to long transverse coherence lifetimes (^29^Si *T*_2_′_CPMG_ of 78 ms ± 2 ms) so that an additional substantial sensitivity gain (more than a factor 7 here) can be obtained by Carr–Purcell–Meiboom–Gill (CPMG)^18b,21^ acquisition. The dependence of silicon-29 *T*_2_′_CPMG_ *versus* the spinning frequency together with the CPMG free induction decays at 10 and 40 kHz MAS are shown in Fig. 4. At 40 kHz, *T*_2_′_CPMG_ values three times longer (78 ms) than those measured at 10 kHz (23 ms) are observed.

Thus, we note that despite the presence of the paramagnetic radical in the polarizing solution, faster MAS frequencies dramatically help to increase the transverse coherence lifetimes of the surface species, opening new avenues for DNP enhanced solid-state NMR spectroscopy. A two-dimensional (2D) DNP-enhanced ^1^H–^29^Si heteronuclear correlation (HETCOR) spectrum recorded with CPMG acquisition in the direct dimension and at 40 kHz MAS is presented in Fig. S14 (ESI†). The sensitivity enhancement obtained with the 1.3 mm DNP probe is also sufficient to record ^1^H–^13^C multi-dimensional experiments on surface species at natural isotopic abundance, as illustrated in Fig. 3b that shows a ^1^H–^13^C HETCOR spectrum of **I** at 40 kHz spinning frequency. All the expected correlations are observed in this spectrum recorded in about 10 hours and on only 2.2 mg of sample.

## Conclusions

In conclusion, using a 1.3 mm DNP probe operating at 18.8 T and ∼110 K, we report here the first experimental investigation of CE DNP at very fast sample spinning (up to 40 kHz MAS). We show that high overall enhancement factors (of about 60) can be obtained on bulk solutions. Despite increased quenching effects at higher spinning rates, fast spinning is shown to result in long coherence lifetimes, and is shown to enable rapid acquisition of multi-dimensional spectra using, for example, efficient CPMG schemes.
