# Supplementary material for: Dynamic nuclear polarization at 40 kHz magic angle spinning
Source: Phys Chem Chem Phys. 2016 Mar 14;18(15):10616–22. doi: 10.1039/c6cp00839a (PMC5048395; doi:10.1039/c6cp00839a)
Supplement: Supplementary file 1 [file CP-018-C6CP00839A-s001.pdf]

Electronic Supplementary Material (ESI) for Physical Chemistry Chemical Physics.
This journal is © the Owner Societies 2016

Supplementary Information

Dynamic Nuclear Polarization at 40 kHz Magic Angle Spinning

Sachin R. Chaudhari,[1] Pierrick Berruyer,[1] David Gajan,[1] Christian Reiter,[2] Frank Engelke,[2] Daniel L. Silverio,[3] Christophe Copéret,[3] Moreno Lelli,[4] Anne Lesage,[1] Lyndon Emsley.[5]

[1]Institut de Sciences Analytiques, Centre de RMN à Très Hauts Champs, Université de Lyon (CNRS/ENS Lyon/UCB Lyon 1), 69100 Villeurbanne, France; [2]Bruker Biospin, 76287 Rheinstetten, Germany; [3]ETH Zürich, Department of Chemistry, Vladimir Prelog Weg 1-5, CH–8093 Zürich, Switzerland; [4]University of Florence, Chemistry Department, Magnetic Resonance Center, 50019 Sesto Fiorentino (FI), Italy [5]Institut des Sciences et Ingénierie Chimiques, Ecole Polytechnique Fédérale de Lausanne (EPFL), CH-1015 Lausanne, Switzerland.

S1. Experimental details
S2. Details for the enhancement and contribution factor Q measurements
S3. Table S1. Experimental NMR parameters for ^{1}H echo-detected experiments, ^{1}H T_{DNP} and CP experiments for measurement of DNP enhancements.
S4. Table S2. Experimental NMR parameters for ^{1}H echo-detected experiments, ^{1}H T_1 and CP experiments for measurements of contribution factor.
S5. Table S3. Summary of DNP Solid-state NMR experiments on the ^{13}C-labelled proline and glycine samples.
S6. Illustration of DNP enhancement at different spinning frequency.
S7. ^{13}C CPMAS NMR spectra of uniformly ^{13}C-labeled proline (0.25 M proline in glycerol-d_8/D_2O/H_2O, 60:30:10 volume ratio containing 10 mM AMUPOL) at 18.8 T and 8 kHz magic angle spinning frequency using 3.2 mm sapphire rotor.
S8. ^{13}C CPMAS NMR spectra of uniformly ^{13}C-labeled proline (0.25 M proline in glycerol-d_8/D_2O/H_2O, 60:30:10 volume ratio containing 10 mM AMUPOL) at 18.8 T and 20 kHz magic angle spinning frequency using 1.3 mm sapphire rotor.
S9. Details on the synthesis and characterization of **I**
S10. Illustration of DNP enhancement on **I** at different spinning frequency using ^{1}H-NMR.
S11. Experimental Details for DNP ^{1}H-^{29}Si CPMG MAS experiments.
S12. ^{29}Si CP-CPMG MAS spectrum of **I** recorded with 120 echoes
S13. Contour plot of a two-dimensional ^{1}H-^{29}Si spectrum with CPMG acquisition in the direct dimension of **I** recorded with DNP at 18.8 T (800 MHz) and 40 kHz MAS.
S14. References

Experimental Details: The Glycerol-d_8, D_2O, ^{13}C-labelled proline and glycine were purchased from Eurisotop (solvents) and from CIL (amino acids). The AMUPol and TEKPol biradicals were prepared according to the synthesis previously reported.[1] 1-Phenylimidazole was purchased from Aldrich and used as received.

DNP-NMR Methods: MAS DNP NMR experiments were performed on a Bruker Avance III 800 MHz wide bore spectrometer, equipped with a triple resonance 1.3 mm low-temperature CP-MAS probe. DNP was achieved by irradiating the sample with high-power microwaves (16 W at the probe) at a frequency of 527 GHz, generated by a gyrotron that was operating continuously during the DNP experiments (stability of better than ±1%). Zirconia rotors were used for all experiments (with an exception for the experiment reported in Figure S8). Spinning frequencies were regulated to ± 20 Hz for all the experiments. A small amount of KBr was added to the bottom of rotor over which silicon plug was inserted to monitor sample temperature by measuring the ^{79}Br T_1 relaxation time.[2] A constant sample temperature of 115 K ± 3 was kept constant over the whole spinning range all experiments.

One-dimensional (1D) ^{1}H direct excitation experiments were acquired with a rotor synchronized spin echo sequence in order to suppress the background signal of the probe. $\pi/2$ and π pulses of 2.5 µs and 5.0 µs (100 kHz) were used respectively. The echo delays (τ) were set to one rotor period. ^{1}H longitudinal relaxation times (T_1) and DNP build-up times (T_{DNP}) were measured with a standard saturation recovery sequence followed by an echo period before signal acquisition (saturation block—τ_{recovery}—$\pi/2$ —τ— π —τ—acquisition) under microwave off and microwave on conditions, respectively. Conventional cross-polarization (CP) experiments were used for the acquisition of the 1D ^{13}C spectra. The ^{1}H and ^{13}C chemical shifts were referenced to TMS at 0 ppm.

Details for the enhancement and contribution factor θ measurements

Measurement of the observed DNP enhancement $\varepsilon_{13C\ CP}$

A 0.25 M ^{13}C-labelled proline in glycerol-d_8/D_2O/H_2O (60/30/10; v/v/v) containing 10 mM AMUPol was prepared. The DNP enhancement factor $\varepsilon_{13C\ CP}$ was obtained from ^{13}C CPMAS experiments, and calculated as the peak intensity ratio measured with and without μw irradiation. The reported enhancements correspond to the mean value of the enhancement factors measured on the 5 carbon-13 resonances of proline. The recycle delay between scans was 5 s. The cross-polarization step was achieved with a proton radio-frequency (RF) field of 81 kHz and the ^{13}C RF field was adjusted for optimal signal for each spinning frequency (see Table S1). Other experimental parameters are reported in Table S1.

Measurement of the contribution factor θ

The contribution factor was measured as described in reference[3], using samples of 2-^{13}C-labelled glycine in bulk water/glycerol solutions containing or not AMUPOL (at a concentration of 10 mM) and calculated as the ratio of the integrated intensities (II) per unit of mass of the CH_2 resonance in ^{13}C CPMAS spectra recorded in the absence of μw irradiation for solutions with and without AMUPOL:

$$\theta = \frac{II_{[AMUPOL]}}{II_{[undoped\ solution]}}$$

Recycle delays of 500 s and 50 s were used to allow for complete signal relaxation for the solution without and with AMUPol respectively. The other experimental details are given in Table S2.

Measurement of the overall sensitivity enhancement factor $\Sigma_{C\ CP}$

The overall DNP enhancement can be calculated following equation[3]:

$$\Sigma_{C\ CP} = (\varepsilon_{C\ CP})(\theta_{C\ CP})\sqrt{T_1/T_{DNP}}$$

Overall DNP enhancement factor including Boltzmann enhancement ($\Sigma^{\dagger}{}_{C\ CP}$)[3] can be calculate as:

$$\Sigma^{\dagger}{}_{C\ CP} = (298\ K/105\ K)(\varepsilon_{C\ CP})(\theta_{C\ CP})\sqrt{T_1/T_{DNP}}$$

Table S1. Experimental NMR parameters used for the acquisition of the ^{13}C CPMAS experiments on the ^{13}C-labelled proline solutions.

^{13}C Cross Polarization	
^{1}H $\pi/2$ pulse	2.5 μs
Contact time (τ_{CP})	2.0 ms
^{1}H RF field for decoupling	100 kHz with SPINAL-64
Linear Ramp	70 % to 100 %
Acquisition time	10.7 ms
Complex point	2048
Exponential window function	400 Hz

MAS (kHz)	CP power	
	^{1}H rf field during contact pulse (kHz) at 100% of power of the ramp	^{13}C rf field during contact pulse (kHz)
5	81	76
10	81	68
15	81	76
20	81	52
25	81	39
30	81	50
35	81	28
40	81	28

Table S2. Experimental NMR parameters used for the acquisition of the ^{13}C CPMAS and of the ^{1}H T_1 and ^{1}H T_{DNP} experiments recorded on the ^{13}C-labelled glycine solutions.

^{1}H 1D echo-detected	
π/2 and π pulses	2.5 μs and 5.0 μs
Acquisition time	10.2 ms
Complex points acquired	2048
Exponential window function	200 Hz
^{1}H T_{DNP}	
recycle delay (s)	0.1 s
π/2 and π pulses	2.5 μs and 5.0 μs
Acquisition time	10.2 ms
Complex points acquired	4096
Exponential window function	200 Hz
^{13}C Cross Polarization	
^{1}H π/2 pulse	2.5 μs
Contact time (t_{CP})	0.5 ms
^{1}H rf field during decoupling	100 kHz SPINAL-64 decoupling
Linear Ramp	70 % to 100 %
Acquisition time	10.2 ms
Complex point	1184
Exponential window function	400 Hz

MAS (kHz)	CP Power	
	^{1}H rf field during contact pulse at the top of the ramp (kHz)	^{13}C rf field during contact pulse (kHz)
5	81	76
10	81	76
15	81	52
20	81	52
25	81	37
30	81	37
35	81	37
40	81	45

Table S3. Summary of the DNP enhanced NMR experiments recorded on the ^{13}C-labelled proline and glycine samples.

MAS (kHz)	Average DNP Enhancement for proline Sample ($\varepsilon_{13C\ CP}$)	T_{DNP} for proline Sample (s)	Enhancement for glycine Sample ($\varepsilon_{13C\ CP}$)	T_1 for glycine Sample (s)	T_{DNP} for glycine Sample (s)	Quenching (scaled by sample mass of the solution with or without the radical (1.595 versus 1.947 mg)
0.4	25 ± 3	_	16± 2	_	-	_
5	52 ± 5	_	35 ± 4	29± 0.2	5.1± 0.2	0.85 ± 0.03
10	58 ± 6	7.2	40 ± 4	_	5.2± 0.2	0.81 ± 0.03
15	66 ± 7	_	37 ± 4	_	5.3± 0.2	0.70 ± 0.03
20	61 ± 6	7.8	39± 4	29± 0.2	5.5± 0.2	0.71 ± 0.03
25	60 ± 6	_	40 ± 4	_	5.8± 0.2	0.58 ± 0.03
30	79 ± 8	8.5	41 ± 4	30± 0.2	6± 0.2	0.58 ± 0.03
35	63 ± 6	_	44 ± 4	_	6.4± 0.2	0.48 ± 0.04
40	56 ± 5	9.2	38 ± 4	29.3±0.2	6.6± 0.2	0.46 ± 0.04

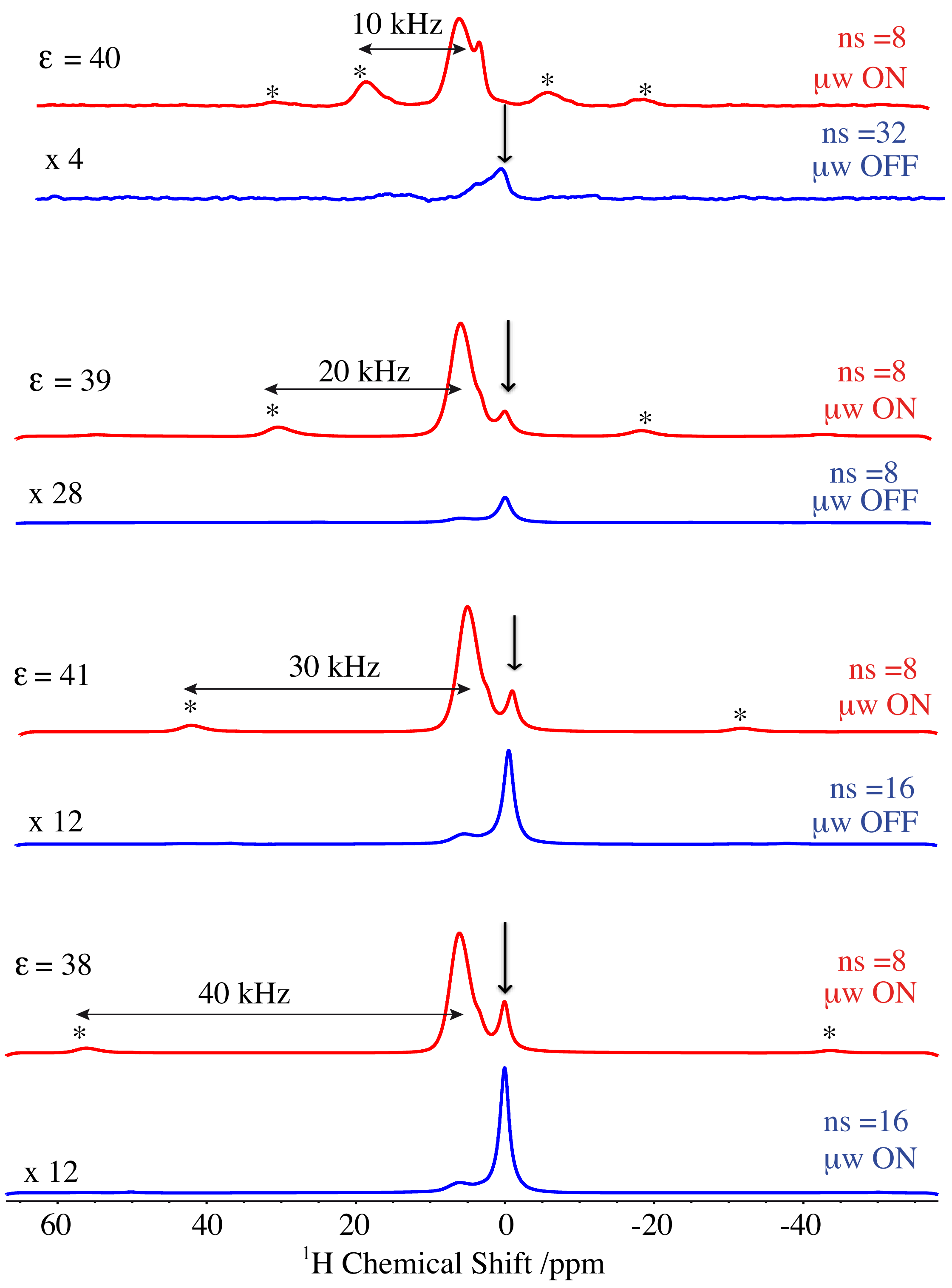

Figure S6: One-dimensional proton spin echo experiments recorded on the glycine solution at different spinning frequencies. Asterisks denote spinning sideband signals. Vertical arrow indicates the ^{1}H resonance of the silicon plug. The enhancements were calculated from ^{13}C-CP MAS).

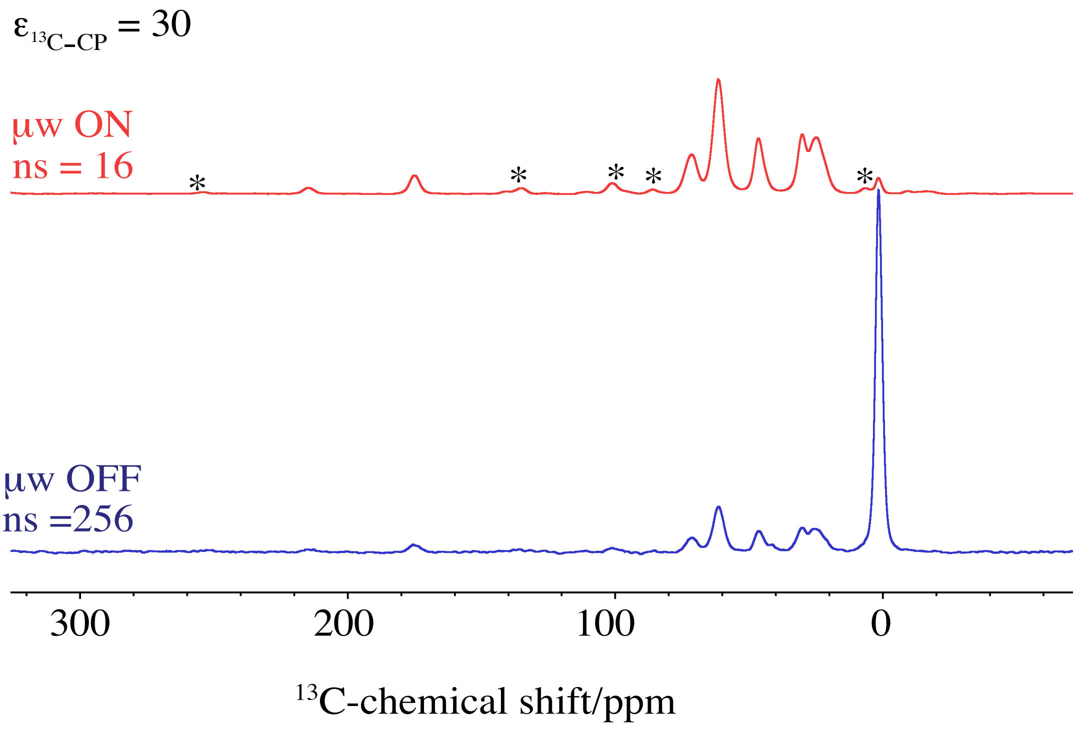

Figure S7: ^{13}C CPMAS NMR spectra of uniformly ^{13}C-labeled proline (0.25 M proline in glycerol-d_8/D_2O/H_2O, 60:30:10 volume ratio containing 10 mM AMUPOL) at 18.8 T and 8 kHz magic angle spinning frequency using 3.2 mm sapphire rotor. The spectra were recorded with (top spectrum) or without (bottom spectrum) μw irradiation to induce DNP. Sample temperature was 131 K (±3) upon mw irradiation as measured from the ^{79}Br T_1 relaxation time of solid KBr crystals.

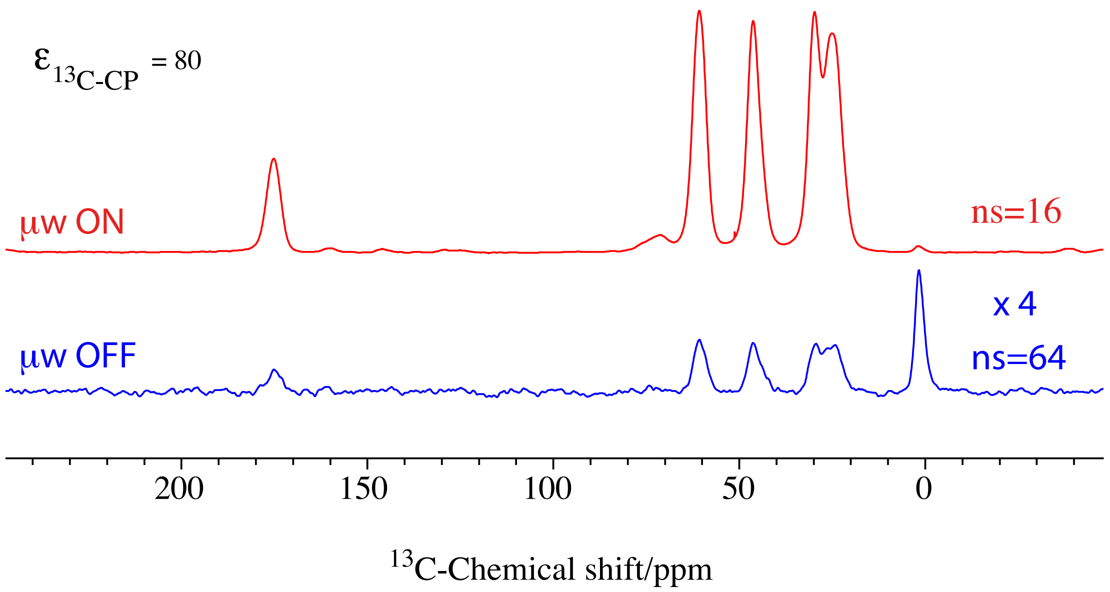

Figure S8: ^{13}C CPMAS NMR spectra of uniformly ^{13}C-labeled proline (0.25 M proline in glycerol-d_8/D_2O/H_2O, 60:30:10 volume ratio containing 10 mM AMUPOL) at 18.8 T and 20 kHz magic angle spinning frequency using 1.3 mm sapphire rotor. The spectra were recorded with (top spectrum) or without (bottom spectrum) μw irradiation to induce DNP.

Details on the synthesis and characterization of the material

All synthetic reactions were carried out with glassware and Telflon stir bars that were oven-dried (150 ºC for at least two hours) and took place in an Ar atmosphere unless otherwise noted. All solvents for synthetic reactions were purified using an MBraun SPS-800 solvent purification system unless otherwise noted. All work-up procedures were carried out with unpurified solvents and reagents without taking special precautions to exclude air and moisture, unless otherwise noted. All SBA-type materials were dried for at least five hours under a vacuum of less than 10-4 Torr at 135 ºC using a temperature ramp of 1 ºC/min from room temperature (~23 ºC).

Elemental Analysis:
Elemental analysis was carried out by Mikroanalytisches Labor Pascher.

Nitrogen Adsorption-Desorption:
The Nitrogen adsorption and desorption measurements were carried out at 77 K using a BELSORB-Mini from BEL-JAPAN. Before N2 adsorption, the samples were degassed at less than 10-4 Torr at 408 K for at least 5 hours. Both the pore volume and the peak pore diameter was calculated using the Barrett–Joyner–Halenda (BJH) method. The specific surface area (SBET) was calculated according to the Brunauer–Emmett–Teller (BET) equation.

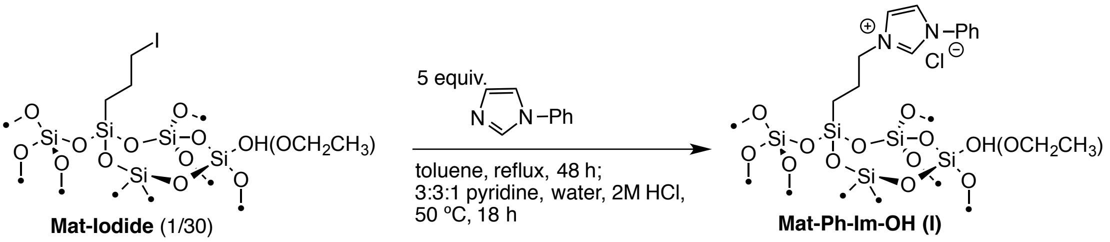

Synthesis of Material Ph-Im-OH:
In an Ar-filled glovebox, a 100 mL round-bottomed flask equipped with a stir bar is charged with 1.5 g of 1/30 Mat-Iodide1 (~0.74 mmol), sealed with a rubber septum and electrical tape, and brought into a fume hood. A needle connected to a flow of Ar is attached to the vessel and, via syringe, 30 mL of toluene is added followed by 0.47 mL (0.54 g, 3.7 mmol) of 1-phenylimidazole. The rubber septum is quickly exchanged for a reflux condenser capped with a rubber septum. The reaction mixture is then heated to reflux and allowed to stir for 48 h while being protected from light with aluminum foil.
After allowing the reaction mixture to cool to room temperature, the resulting pale yellow solid is collected on a porosity 3 fritted funnel and is washed with dichloromethane (3 x 30 mL). The solid is then transferred to a 100 mL round-bottomed flask containing a stirring mixture of pyridine, deionized water, and 2M aqueous HCl (18:18:6 mL, respectively), and the reaction vessel is capped with a rubber septum contained a vent needle. The reaction mixture is heated to 50 ºC and allowed to stir for 18 h.

[1] J. Alauzun, A. Mehdi, C. Reyé and R. Corriu, *New J. Chem.* **2007**, *31*, 911–915.

After allowing the reaction mixture to cool to room temperature, the resulting pale yellow solid is collected on a porosity 3 fritted funnel and is washed with deionized H2O (3 x 30 mL), acetone (3 x 30 mL), and diethyl either (3 x 30 mL). The solid is then dried for 12 hours under a vacuum of less than 10-4 Torr at 135 ºC using a temperature ramp of 1 ºC/min from room temperature (~23 ºC) and brought into an Ar-filled glovebox. Following drying, 1.2 g of an off-white solid was obtained. Elemental analysis: C = 6.28%wt; H = 1.90%wt; N = 1.37%wt. C/N ratio obtained: 5.9 (expected 6.0). Nitrogen adsorption-desorption: Specific surface area (BET) = 688 m2/g; Pore volume (BJH) = 1.2 cm3/g; Peak pore diameter (BJH) = 8.1 nm.

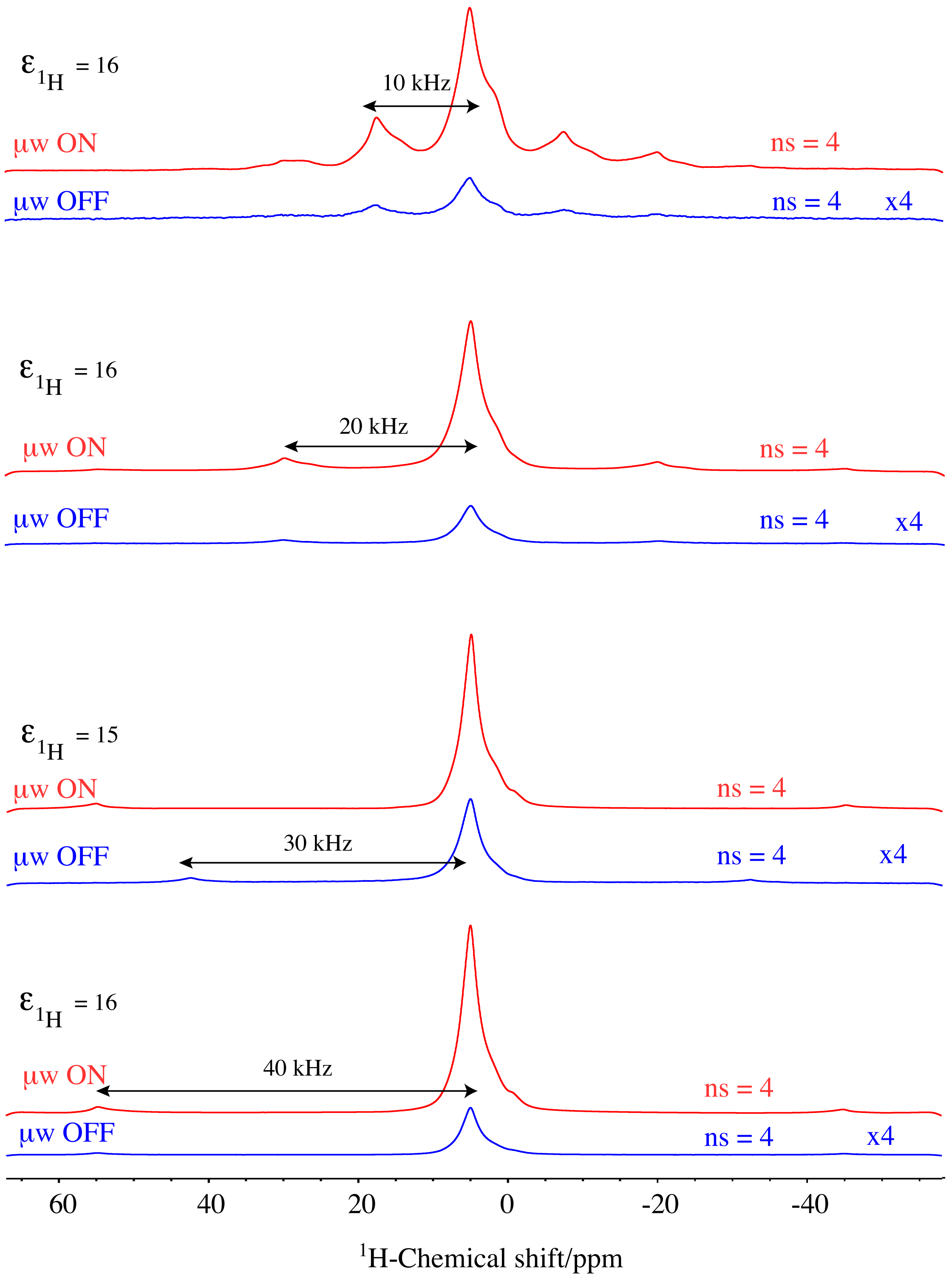

Figure S11: One-dimensional proton spin echo experiments recorded on **I** at different spinning frequencies. Double-headed arrow denotes spinning sideband signals. The structure of the material is identical to that of Figure 3 (main text).

Table S1. Summary of the DNP enhanced ^{1}H-^{29}Si CP/CPMG experiments recorded on sample **I** at different MAS frequencies.*

MAS (kHz)	Number of Meiboom-Gill loops [N]	^{29}Si rf field during CP (kHz)	$^{a}T_2$' (ms)	$^{b}T_2$' (ms)
40	60	18	78	40
30	40	18	70	-
20	60	39	47	-
10	40	41	23	22

Other experimental Details
^{29}Si 180° pulse length [π] (6 μ s)
^{1}H 90° pulse lenght [π /2] (2.5 μ s)
Dwell time (8.4 μ s)
^{1}H rf field amplitude during CP (71 kHz) **
Spectral width = 375 ppm
Number of scans = 256
Recycle delay = 4 s
CP contact time = 3 ms

* T_2' are silicon-29 T_2' measured by fitting the intensity of the echo tops extracted from the CP/CPMG FIDs to a monoexponential decay function: $S(t) = S_0 \times \exp(-t/T_2')$. ^{a}SPINAL-64 heteronuclear decoupling[4] was applied during t_2 with an rf amplitude of 130 kHz; ^{b}SPINAL-64 heteronuclear decoupling[4] was applied during t_2 with an rf amplitude of 100 kHz

** A ramp from 70 to 100 % was used on the proton channel. The rf field indicated here is the field at the top of the ramp.

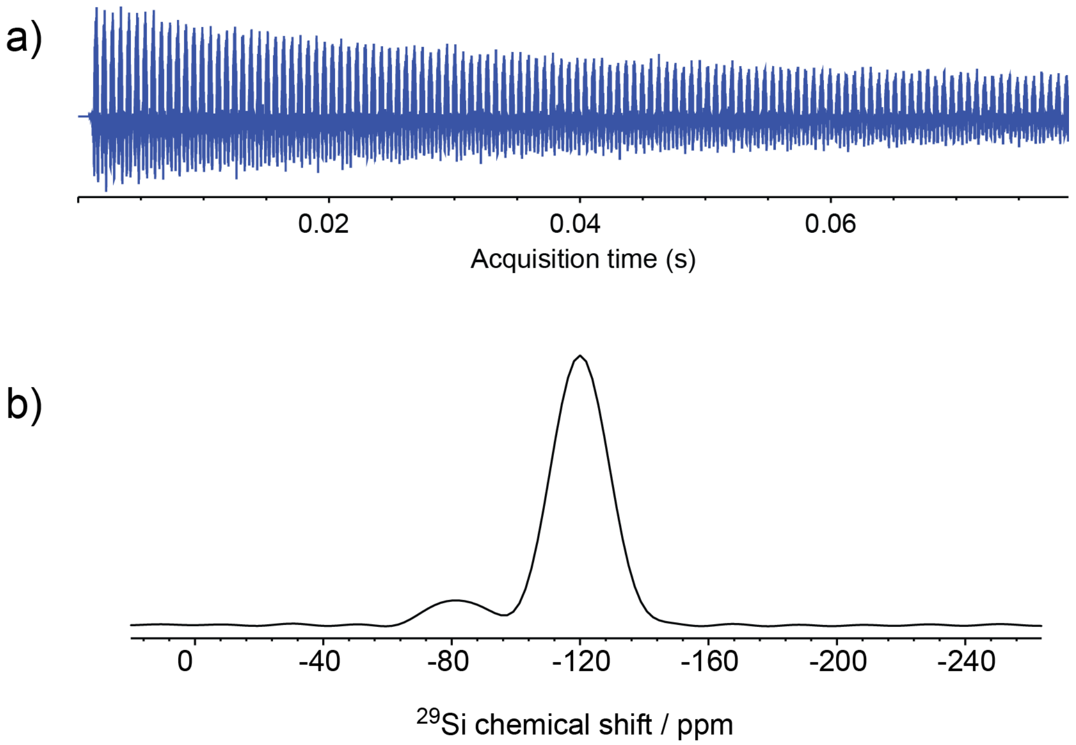

Figure S13: a) Free induction decays of a ^{1}H-^{29}Si CP/CPMG experiment recorded at 40 kHz MAS frequency. SPNAL-64 decoupling at 130 kHz RF field was applied during acquisition. A total of 120 echoes were acquired corresponding to an acquisition time of 80 ms. The other experimental details were the same as those given in the caption of Fig. 3a. b) The reconstructed CP-CPMG spectrum obtained by adding up the whole echoes of the FIDs in the time domain, followed by Fourier transform and application of a first-order phase correction.

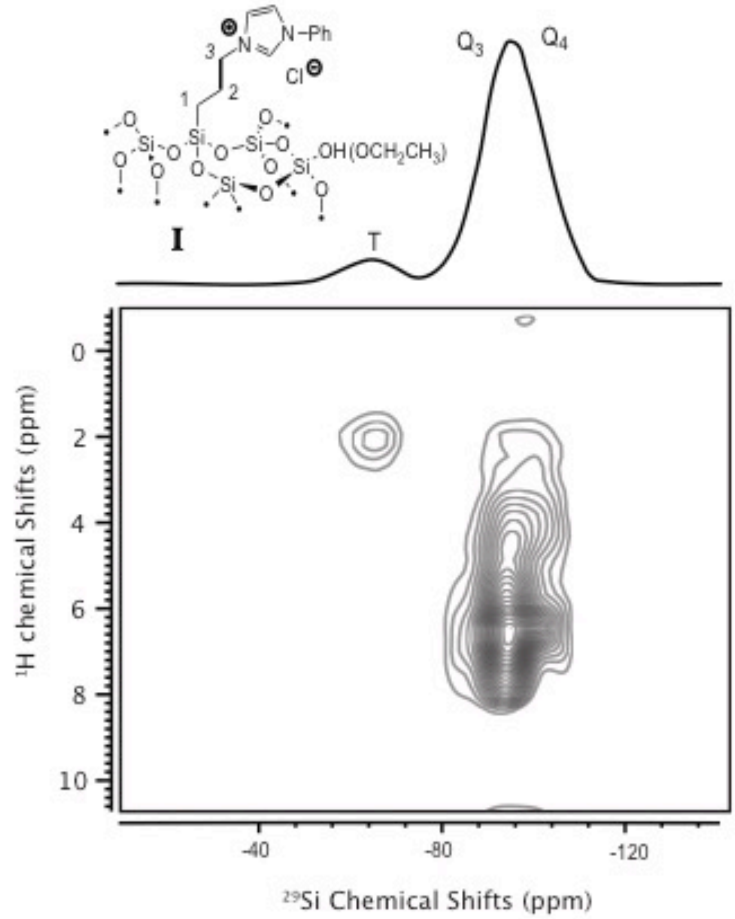

Figure S14: Contour plot of a two-dimensional ^{29}Si DNP-SENS HETCOR-CPMG spectrum of **I**, recorded at 18.8 T (800 MHz) and 40 kHz MAS. The structure of the material is identical to that of **I** in Figure 3 (main text). The sample was impregnated with a solution of 10 mM AMUPOL in 90:10 D_2O/H_2O, T ≈ 115 K. During direct acquisition, SPINAL-64 decoupling[4] was applied with a rf amplitude of 130 kHz. A total of 42 echoes were acquired. During t_1, eDUMBO-1_{22} [5] homonuclear decoupling was applied with an rf amplitude of 150 kHz. A scaling factor of 0.56 was applied to correct the ^{1}H chemical shift scale.[5] The total experimental time was 11.5 hours. The CPMG spectrum is shown in its echo reconstructed form and was obtained by summing the whole echoes of the FIDs in the direct time domain, followed by Fourier transform.

References:

[1] a) C. Sauvee, M. Rosay, G. Casano, F. Aussenac, R. T. Weber, O. Ouari and P. Tordo, *Angew. Chem. Int. Ed. Engl.* **2013**, *52*, 10858-10861; b) A. Zagdoun, G. Casano, O. Ouari, M. Schwarzwalder, A. J. Rossini, F. Aussenac, M. Yulikov, G. Jeschke, C. Coperet, A. Lesage, P. Tordo and L. Emsley, *J. Am. Chem. Soc.* **2013**, *135*, 12790-12797.

[2] K. R. Thurber and R. Tycko, *J. Magn. Reson.* **2009**, *196*, 84-87.

[3] A. J. Rossini, A. Zagdoun, M. Lelli, D. Gajan, F. Rascón, M. Rosay, W. E. Maas, C. Copéret, A. Lesage and L. Emsley, *Chem. Sci.* **2012**, *3*, 108-115.

[4] B. M. Fung, A. K. Khitrin and K. Ermolaev, *J. Magn. Reson.* **2000**, *142*, 97-101.

[5] B. Elena, G. d. Paëpe and L. Emsley, *Chem. Phys. Lett.* **2004**, *398,* , 532–538.
